# Supplementary material for: Paranormal belief, psychopathological symptoms, and well-being: Latent profile analysis and longitudinal assessment of relationships
Source: PLoS One. 2024 Mar 6;19(3):e0297403. doi: 10.1371/journal.pone.0297403 (PMC10917279; doi:10.1371/journal.pone.0297403)
Supplement: Attachment — Submitted filename: Response to Reviewers.docx [file pone.0297403.s001.docx]

Paranormal belief, psychopathological symptoms, and well-being: Latent profile analysis and longitudinal assessment of relationships

PLOS ONE


Please include the following items when submitting your revised manuscript:

- A rebuttal letter that responds to each point raised by the academic editor and reviewer(s). You should upload this letter as a separate file labeled 'Response to Reviewers'.
- A marked-up copy of your manuscript that highlights changes made to the original version. You should upload this as a separate file labeled 'Revised Manuscript with Track Changes'.
- An unmarked version of your revised paper without tracked changes. You should upload this as a separate file labeled 'Manuscript'.

If you would like to make changes to your financial disclosure, please include your updated statement in your cover letter. Guidelines for resubmitting your figure files are available below the reviewer comments at the end of this letter.

If applicable, we recommend that you deposit your laboratory protocols in protocols.io to enhance the reproducibility of your results. Protocols.io assigns your protocol its own identifier (DOI) so that it can be cited independently in the future. For instructions see: [https://journals.plos.org/plosone/s/submission-guidelines#loc-laboratory-protocols](https://protect-eu.mimecast.com/s/6GkvCZw6liGlE9EFjAXdh?domain=journals.plos.org). Additionally, PLOS ONE offers an option for publishing peer-reviewed Lab Protocol articles, which describe protocols hosted on protocols.io. Read more information on sharing protocols at [https://plos.org/protocols?utm_medium=editorial-email&utm_source=authorletters&utm_campaign=protocols](https://protect-eu.mimecast.com/s/jhfHC1YwmIR31N1TpV6PD?domain=plos.org).

We look forward to receiving your revised manuscript.

Kind regards,

Giulia Prete
Academic Editor
PLOS ONE

Journal Requirements:

When submitting your revision, we need you to address these additional requirements.

1. Please ensure that your manuscript meets PLOS ONE's style requirements, including those for file naming. The PLOS ONE style templates can be found at

[https://journals.plos.org/plosone/s/file?id=wjVg/PLOSOne_formatting_sample_main_body.pdf](https://protect-eu.mimecast.com/s/rdaBC2gkncj1vzvi22a2-?domain=journals.plos.org) and 
[https://journals.plos.org/plosone/s/file?id=ba62/PLOSOne_formatting_sample_title_authors_affiliations.pdf](https://protect-eu.mimecast.com/s/DIbCC3j1ouBqwzwIQ7Bxt?domain=journals.plos.org)

2. Note from Emily Chenette, Editor in Chief of PLOS ONE, and Iain Hrynaszkiewicz, Director of Open Research Solutions at PLOS:

Did you know that depositing data in a repository is associated with up to a 25% citation advantage ([https://doi.org/10.1371/journal.pone.0230416](https://protect-eu.mimecast.com/s/q_pVC4kxpUogyKyTMyqI8?domain=doi.org))? If you’ve not already done so, consider depositing your raw data in a repository to ensure your work is read, appreciated and cited by the largest possible audience. You’ll also earn an Accessible Data icon on your published paper if you deposit your data in any participating repository ([https://plos.org/open-science/open-data/#accessible-data](https://protect-eu.mimecast.com/s/VGnmC5lyqcvEoLotNsAEg?domain=plos.org)).

3. We note that the grant information you provided in the ‘Funding Information’ and ‘Financial Disclosure’ sections do not match.

When you resubmit, please ensure that you provide the correct grant numbers for the awards you received for your study in the ‘Funding Information’ section.

4. We note that you have stated that you will provide repository information for your data at acceptance. Should your manuscript be accepted for publication, we will hold it until you provide the relevant accession numbers or DOIs necessary to access your data. If you wish to make changes to your Data Availability statement, please describe these changes in your cover letter and we will update your Data Availability statement to reflect the information you provide.

5. Please upload a copy of Figure 1 and 2 to which you refer in your text on pages 32-33. If the figure is no longer to be included as part of the submission please remove all reference to it within the text.

6. We note you have included a table to which you do not refer in the text of your manuscript. Please ensure that you refer to Table 1 and 2 in your text; if accepted, production will need this reference to link the reader to the Table.

7. Please include captions for your Supporting Information files at the end of your manuscript, and update any in-text citations to match accordingly. Please see our Supporting Information guidelines for more information: [http://journals.plos.org/plosone/s/supporting-information](https://protect-eu.mimecast.com/s/DxB2C6m8rcDzEYEU2i8t0?domain=journals.plos.org).

8. Please upload a copy of Supporting Information Table 1 & 2 which you refer to in your text on pages 14-15.

9. Please review your reference list to ensure that it is complete and correct. If you have cited papers that have been retracted, please include the rationale for doing so in the manuscript text, or remove these references and replace them with relevant current references. Any changes to the reference list should be mentioned in the rebuttal letter that accompanies your revised manuscript. If you need to cite a retracted article, indicate the article’s retracted status in the References list and also include a citation and full reference for the retraction notice.

**Additional Editor Comments:**

Both Reviewers stressed that it is a bit unexpected to define this study as a longitudinal study because different measures are collected across time. I agree with this idea and would like to invite you to better define this point, tigether with the other points raised by Reviewer 1. 


[Note: HTML markup is below. Please do not edit.]

**Reviewers' comments:** Reviewer's Responses to Questions

**General Comments**

With formatting and referencing, we apologise that the submitted version of the manuscript did not adhere to the conventions. On checking, it appears that we used a version where the referencing did not wholly align with the numbering system in text. Additionally, within the reference section citations were a mixture of Vancouver and APA. This was due to the fact that the Authors use APA as a default and the conversion process was only partially implemented. The resubmitted manuscript contains the aligned, amended, and updated references as requested.

**Reviewer #1:**

**Comment**

• Authors are absolutely correct that most paranormal studies represent a single moment in time. There are virtually no studies that look at paranormal belief across time.

**Response**

Thank you for acknowledging this point.
**Comment**

• Great introduction that covers the basis and details of “believers” and their brain function.

**Response**

We made every attempt to include a breadth of pertinent research and synthesise it around prevailing design, methodological, and analytical limitations.

**Comment**
• State upfront that this study is “exploratory” and that “precise hypotheses cannot be stated”.

**Response**

Yes – the approach due to novelty and complexity was exploratory in nature.

**Comment**

• Participants are self-enrolled via a website for people interested in participating in research of some kind. This could lead to some bias in the participants and will limit the extrapolation ability of this study.
**Response**

This is a common concern. We do check data quality and make comparisons with relevant studies. The provider has an established track record of providing data that is equivalent to that collected via traditional methods. In the context of my previous comments we are highly confident that these data were representative (more so than university-based samples) of general populations.

**Comment**

• Reported the alpha values of the surveys and they were in a decent range showing good reliability on the surveys.

**Response**

Reliability across measures was good as would be expected with a large general sample.

**Comment**
• I think that this paper strives to do a longitudinal study (across time) but they only take the PB survey at the beginning and not each time so the actual belief comparison doesn’t exist throughout the timeline, it is only a comparison of various psychological factors in relation to the original paranormal belief scale. However, if the authors can support that paranormal beliefs don’t really change all that much over the timeline of the study then all of my concerns will be met.
**Response**

This is a good/fair point. The study measures outcomes at various time points to see how these vary as a function of sub-group membership and hence is only partially longitudinal. Noting this we have changed the terminology throughout the paper. That stated, belief in the paranormal over the period measured is unlikely to change significantly. Indeed, we have previously reported that test–retest reliability is in the moderate to high range across time intervals. Also, that the RPBS demonstrates sustained internal consistency. These conclusions align with structural stability.

**Comment**

• Skeptical not Sceptical
**Response**

Have changed throughout. This works better in context, although there are generally cultural variations. We do agree though!

**Comment**

• I found this paper interesting and a new and important piece of information concerning the type of believer personality is within humanity. However, the authors state that this is a longitudinal study, because it has surveys that are given over a time period of several months. However, none of the belief/psychology surveys were repeated so I have some reservations with calling this study a longitudinal study. Instead, this seems to be a study that has numerous qualifiers for various aspects of paranormal belief and psychoses that are associated with a preponderance of belief.

**Response**

Agreed.

**Comment**

In addition, this study links several of the mental factors to a person’s well-being. This is a neat, and unique aspect of this paper. I think it is a great statement that a person’s belief and their mental condition might work together to produce a terrible state of well-being. In contrast, it is great that some of the effects of believing in paranormal might actually offset some bad mental states.

**Response**

Thank you – this was the primary motivation for examining the subject area. Also, to explore nuances and subtleties obscured by reductionist assumptions and resource limitations.

**Comment**

I would like to see this paper accepted, but to have it reworked to eliminate the longitudinal ideas and to have the paper reworked to be more about the connections of various aspects of mental state and paranormal belief.

**Response**

We have amended the references to longitudinal.

**Comment**

If this was truly a longitudinal study then the base scores (paranormal belief) would have been measured throughout the timeline, along with the other mental factors. It is possible for someone’s belief in paranormal subjects to change through time so it would be better to measure if there was any change in this (either all along the timeline, or at the beginning and end).

**Response**

Please see above.

Reviewer #2:

**Comment**

The present longitudinal study investigated whether the membership of profiles derived from PB and psychopathology predicts well-being over time, and it assessed the mediating effect of theoretically important variables (transliminality, happiness orientation, anxious and sceptical attitudes). Results supported the existence of a sophisticated process underpinning the relationship between PB and well-being. Overall, results indicated that PB, in absence of psychopathology, had no significant effect on well-being. "However, authors specify the exploratory nature of the study, so it is not possible to make specific hypotheses.

I would ask the authors to better clarify the concept of longitudinal study because none of the surveys were repeated in the different timepoints.

**Response**

Thanks. These comments align with the other reviewer, and we have removed reference to longitudinal accordingly.

**Comment**
p. 18 close the parenthesis "(e.g., progressive elaboration, [94] are required".

**Response**

Amended.
